# Green Nanocoatings Based on the Deposition of Zirconium Oxide: The Role of the Substrate

**DOI:** 10.3390/ma14041043

**Published:** 2021-02-23

**Authors:** Vitor Bonamigo Moreira, Anna Puiggalí-Jou, Emilio Jiménez-Piqué, Carlos Alemán, Alvaro Meneguzzi, Elaine Armelin

**Affiliations:** 1 Department of Chemical Engineering, Universitat Politècnica de Catalunya, Campus Diagonal Besòs (EEBE), C/Eduard Maristany, 10-14, Building I, 2nd Floor, 08019 Barcelona, Spain; vitor.moreira@upc.edu (V.B.M.); anna.puiggali@upc.edu (A.P.-J.); carlos.aleman@upc.edu (C.A.); 2 Post-Graduation Program in Mining, Metallurgical and Materials Engineering (PPGE3M), Universidade Federal do Rio Grande do Sul (UFRGS), Av. Bento Gonçalves, 9500–91501-970 Porto Alegre, RS, Brazil; meneguzzi@ufrgs.br; 3 Barcelona Research Center for Multiscale Science and Engineering, Universitat Politècnica de Catalunya, Campus Diagonal Besòs (EEBE), C/Eduard Maristany, 10-14, Building I, Basement Floor, 08019 Barcelona, Spain; emilio.jimenez@upc.edu; 4 Department of Materials Science and Engineering, Universitat Politècnica de Catalunya, Campus Diagonal Besòs-EEBE, C/Eduard Maristany, 10-14, Building I, 1st Floor, 08019 Barcelona, Spain; 5 Institute for Bioengineering of Catalonia (IBEC), The Barcelona Institute of Science and Technology, Baldiri Reixac, 10-12, 08028 Barcelona, Spain

**Keywords:** conversion coating, zirconium oxide, aluminum alloys, electro-assisted deposition, metal-oxide interface, nanocoating

## Abstract

Herein, the influence of the substrate in the formation of zirconium oxide monolayer, from an aqueous hexafluorozirconic acid solution, by chemical conversion and by electro-assisted deposition, has been approached. The nanoscale dimensions of the ZrO_2_ film is affected by the substrate nature and roughness. This study evidenced that the mechanism of Zr-EAD is dependent on the potential applied and on the substrate composition, whereas conversion coating is uniquely dependent on the adsorption reaction time. The zirconium oxide based nanofilms were more homogenous in AA2024 substrates if compared to pure Al grade (AA1100). It was justified by the high content of Cu alloying element present in the grain boundaries of the latter. Such intermetallic active sites favor the obtaining of ZrO_2_ films, as demonstrated by XPS and AFM results. From a mechanistic point of view, the electrochemical reactions take place simultaneously with the conventional chemical conversion process driven by ions diffusion. Such findings will bring new perspectives for the generation of controlled oxide coatings in modified electrodes used, as for example, in the construction of battery cells; in automotive and in aerospace industries, to replace micrometric layers of zinc phosphate by light-weight zirconium oxide nanometric ones. This study is particularly addressed for the reduction of industrial waste by applying green bath solutions without the need of auxiliary compounds and using lightweight ceramic materials.

## 1. Introduction

Circular economy, life-cycle assessment, reduction of fuel consumption, reduction of industry manufacturing costs, all together drive forces to the development of environmentally friendly and lightweight new materials and devices for automotive and aerospace industry applications. Within this context, old and expensive metal pre-treatment processes, such as iron and tri-cation zinc phosphating, and green and yellow chromate, are moving towards a new generation of cleaning and surface activation processes [1,2]. Phosphating is a widely used chemical pre-treatment process for metal surface protection, actuating as both passivating layer and adhesion promoter for subsequent application of organic coatings, particularly in the automotive and in the aircraft industry [3,4]. Phosphating and chromate technologies have been substituted by chromium-free and hybrid pre-treatment coatings [5,6,7,8]. The urge for innovation in such processes and, particularly, in the replacement of hazardous substances (with potential toxicity for human life) motivate extensive research on various alternatives, among which titanium and zirconium fluorinated-complex are recognized as one of the most promising pre-treatment coatings for body car protection, aerospace alloys, coil coatings, and other structural equipment [9,10].

Zirconium-based chemical conversion coating (Zr-CCC) has been widely used as chemical pre-treatment by dip-coating deposition and under cathodic electro-dip primer formation, mainly for the replacement of tri-cation zinc phosphate pre-treatments in the automotive original equipment manufacturers (OEM) market [11]. Such coatings, with specific weight of only 0.01 to 0.02 g/m^2^, are extremely thin and represent a great advantage of cost and light-weighting final material for the producers [1]. The effectiveness of the dip-coating process by immersion of the metal substrate in hexafluorozirconic-acid-based (H_2_ZrF_6_-based) solution is pH limited and the homogeneity of the obtained films is sometimes poor [12]. Previous works have demonstrated that the incorporation of auxiliary additives in the bath solutions, like copper (II), nickel (II), nitrate (NO_3_^−^) and hydrogen phosphate (HPO_4_^2−^) ions, are necessary to further improve the barrier property and the density of Zr-CCC layer [13,14]. Unfortunately, the addition of auxiliary compounds is not a good option for industrial applications because they may increase the formation of anodic sludge in tanks and the amount of water contaminants, resulting in non-sustainable and non-competitive costs for industry.

There are a lot of examples of the Zr-CCC applications in steel and aluminum alloys, by dip-coating method [9,15,16,17] and a few related to the electro-assisted deposition (EAD) method [18,19]. Unfortunately, a deep understanding of the nanocoating formation and its stability were scarcely approached. For instance, Chen-Wiegart and co-workers [13] developed environmentally friendly Zr-based conversion nanocoatings on low carbon steel by using H_2_ZrF_6_ solutions with very low content (ppm) of cupric nitrate as additive. By using in situ synchrotron X-ray fluorescence microscopy (XRF), they found that the final nanocoating composition mainly consists on ZrO_2_ and Cu_2_O, Cu, and CuF_2_ molecules in format of clusters above the film. Therefore, Cu ions were retained by chemical reaction adsorption and were not leached to the bath solution.

Herein, we describe the controlled deposition of a hydrated zirconium oxide monolayer, free of other metals or additives, in two grades of aluminum substrates: pure Al (AA1100) and Cu-rich alloy (AA2024). The films, which were obtained using the dip-coating (Zr-CCC or simply DC) and the electro-assisted deposition method (Zr-EAD), were fully characterized by microscopy, spectroscopy and electrochemical analyses. A mechanism based on the substrate nature is proposed, corroborating with previous works on CCC treatments. Finally, the barrier properties are also evaluated to determine the nanocoating resistance in electrolyte solution. Considering that H_2_ZrF_6_ is the main component of most Zr-based conversion treatments, this study is appealing to broad applications.

## 2. Materials and Methods

### 2.1. Materials

Hexafluorozirconic acid (H_2_ZrF_6_, 50 % w/w in water) was supplied by Sigma-Aldrich Co. (Madrid, Spain); sodium hydroxide (NaOH pellets), used to regulate the pH of the solution, was purchased from Panreac S.A. (Barcelona, Spain). The industrial alkaline degreaser (Saloclean 667N) was kindly supplied by Klintex Insumos Industriais Ltda. (São Paulo, Brazil). Aluminum plates (standard name AA1100), with rectangular geometry (5.0 × 1.4 × 0.3 cm^3^), and aluminum alloy (standard name AA2024-T3), with disk format (3.5 cm in diameter and 4 mm of thickness) were used as substrates for Zr-CCC and Zr-EAD formation. AA1100 is included in the commercially pure Al series. Its composition was provided by the supplier Irmãos Galeazi Ltda. (São Paulo, Brazil), in weight percentage (in wt. %): Cu (0.34); Fe (0.42); Si (0.19); Mn (<0.01); Ti (<0.02); Zn (<0.01) and balanced aluminum. AA2024-T3 is a structural reinforced aluminum-copper grade with metallurgical temper code T3, which is referred to a solution heat-treated, cold worked and metal naturally aged. The AA2024 bar was supplied by Balumer S.L. (Barcelona, Spain) and its chemical composition is (in wt.%): Cu (4.63); Mg (1.66); Mn (0.55); Fe (0.36); Si (0.31); and balanced to Al. The chemical composition analysis for AA2024 alloy was determined by ICP-AES (spectrometer SPECTROMAXx, Spectro, Cleve, Germany). Indium tin oxide coated polyethylene terephthalate sheets (ITO coated PET sheets, surface resistivity 60 Ω/square, Sigma-Aldrich, Saint Louis, MI, USA) and pure copper plates were used as complementary substrates for specific tests, described in the next sections.

### 2.2. Preparation and Cleaning of Aluminium Substrates

The surfaces of the aluminum alloy substrates were prepared by grinding the surface with silicon carbide paper from #600-grit, down to up #2500-grit. Some samples were cleaned in ultrasound bath with isopropanol, followed by ethanol and, at last, acetone for 5 min at room temperature in each solvent. This procedure was carried out for the production of Raw samples, providing a mild cleaning, aiming for the preservation of the naturally formed aluminum oxide layer (hereafter coded as “Raw”). The rest of the samples were thoroughly washed with water and acetone and immersed in Saloclean 667N degreasing agent (pH 9.4, 70 g/L, 70 °C) for 5 min. Afterwards, the specimens were washed with distilled water in a sonication bath for 5 min, dried under a hot air stream, and stored under vacuum before use. This treatment generated the samples coded as “Bare”, which represents the industrial conditions of metal degreasing before coating deposition.

### 2.3. Zirconium Oxide Nanocoating Depositions (DC and Zr-EAD)

The conversion bath consists of an aqueous solution prepared with 100 mg of H_2_ZrF_6_ in 1 L of water (0.015% *v*/*v*). The pH was adjusted to 3.5 with NaOH solution (1 M). In order to produce the Zr-DC samples, panels were immersed in the conversion solution for 4 min, followed by water rinsing and hot air stream drying (before aging step, described below). By contrary, the Zr-EAD samples were obtained by a potentiostatic electrochemical method. The depositions were carried out with an Autolab PGSTAT302N potentiostat-galvanostat (Ecochimie) equipment, by using a three electrodes cell configuration. The working electrodes consisted of aluminum plates or disks, ITO foils and copper plates; the reference electrode was Ag|AgCl (3 M, saturated); and the counter electrode was composed by graphite bar. The electrolyte used for EAD assays was the same used for the chemical conversion process. The experiments were carried out after open circuit potential (OCP) measurement (30 s), followed by the application of the desired potential during 4 min. The potentials used in this work were −1.0 V_OCP_ and −1.5 V_OCP_, generating the samples named “EAD −1.0 V” and “EAD −1.5 V”, respectively. Such potentials were chosen on the basis of our previous experience [20,21]. Samples coated by both dip-coating and EAD were further post-treated for 2 h at 100 °C, as aging step treatment to achieve the dehydration of the oxide layer and the reduction of defects [22].

In parallel, ITO substrates were sequentially rinsed in deionized water, acetone and ethanol, with each rinsing step being carried out during 5 min, under sonication. After the washing process, the samples were submitted to the ZrO_2_ electro-assisted deposition, with the same procedure and equipment described for the aluminum specimens. Those samples were used for the AFM and SEM measurements, in order to evaluate the isolated effect of EAD in a substrate where the conversion chemical reactions do not occur and to approach the influence of substrate homogeneity and roughness on the Zr-EAD coating. Moreover, more samples were prepared by EAD method, employing longer reaction time (60 min), to achieve thicker films and measure the mechanical properties of the oxide layer by nanoindentation test. In the same way, copper plates were also cleaned and, further DC or EAD methods were applied to compare the effect of Cu metal in the ZrO_2_ production and the stability in electrolyte media.

### 2.4. Physical and Chemical Characterization

Scanning electron microscopy (SEM) studies were conducted with a Focus Ion Beam (FIB) Zeiss Neon 40 instrument (Carl Zeiss, Berlin, Germany). One specimen of each coated substrate was selected for cross-section analysis. The cross-sections were cut using Focused Ion Beam (FIB) with gallium ions. The samples were previously coated with a sputtered carbon layer and received a thin protective layer of platinum (gas injection). First, an electron beam assisted Pt deposition was carried out to achieve Pt layer thickness of about 250 nm. The platinum layer was necessary to obtain smoother cross-sections by protecting the surface against the damage of the Ga ions. An initial coarse milling was carried out at 10 nA, followed by a finer milling at 500 pA, which provided polished cross-sections was performed. The resulting cross-sections were 15 μm long and 3 μm deep. For SEM analysis an electron beam of 5 kV was applied. The thickness of Zr-DC and Zr-EAD coatings was evaluated after 50 measurements using high magnification images. Analysis of variance (one-way ANOVA) was carried out with a confidence level of 95%, followed by a Turkey test for means comparison of the measured thicknesses. Atomic force microscopy (AFM) was conducted to obtain topographic information (2D Phase images) and roughness (3D height images) of the modified surfaces. The experiments were carried out with an AFM Dimension microscope, equipped with NanoScope IV controller and NanoScope Analysis software (Veeco Instruments Inc., New York, NY, USA), under ambient conditions and by using tapping mode. The equipment operated with a frequency of 150 kHz and a with force constant of 5 N/m. The scanning frequency was set between 0.6 and 0.8 Hz and the area of analysis varied with windows of 50 µm^2^ × 50 µm^2^ and 20 µm^2^ × 20 µm^2^. Several measurements were performed on the top of the samples, which produced reproducible images similar to those displayed in this work.

The chemical compositions of the substrates and coatings were determined by X-ray photoelectron spectroscopy (XPS) and energy dispersive X-ray spectroscopy (EDX). XPS analyses were performed in a SPECS system equipped with a high intensity twin anode X-ray source XR50 of Mg/Al (1253 eV/1487 eV) operating with the selected Al anode at 150 W, and by using a Phoibos 150 MCD-9 XP detector (SPECS, Berlin, GmbH). For the preparation of the samples, the bare alloys were first polished, washed three times with isopropanol, ethanol, deionized water and acetone, under ultrasound bath for 5 min, dried under nitrogen flow and vacuum. Afterwards, sputtering of the surfaces with argon gas (2 min) was applied to eliminate the surface contamination by carbon compounds. All spectra were calibrated with the C 1s peak (284.5 eV). The following elements: C 1s, O 1s, Al 2p, Zr 3d, and Cu 2p were analyzed for the high-resolution peaks and plotted after deconvolution.

Water contact angle (WCA) measurements were carried out with an OCA 15EC instrument (DataPhysics Instruments, Filderstadt, GmbH), using the sessile droplet method at room temperature, to evaluate the wettability changes of the modified surfaces. Data were recorded after the water droplet deposition (0.5 μL) and stabilization (30 s), using SCA 20 software, and are reported as an average of ten independent measures for each sample.

The hardness and elastic modulus of thin Zr-EAD films were measured using an MTS Nanoindenter XP instrument (Nanoinstruments Innovation Center, Oak Ridge, TN, USA), with a Berkovich diamond tip calibrated with fused silica standard. The results were analyzed with the Oliver and Pharr method [23,24]. The sample prepared on ITO substrate, coated by EAD technique at −1.0 V during 60 min, was used as model to estimate the mechanical properties of the film. Long generation time was necessary to produce thick coatings in order to obtain reliable values of mechanical properties under nanoindentation forces.

### 2.5. Electrochemical Characterization of Zirconium Oxide Nanocoating

The potentiodynamic and electrochemical impedance spectroscopy (EIS) studies were performed in an Autolab PGSTAT302N (Eco-Chemie Methrom Autolab B.V., Utrecht, The Netherlands) potentiostat-galvanostat (Ecochemie) equipment. The electrochemical cell consisted of three electrodes, having the sample set as the working electrode, a platinum wire as counter electrode and a Ag|AgCl (KCl, 3 M) as reference electrode. The tested area was 0.785 cm^2^ and the electrolyte used was 0.05 M NaCl aqueous solution. The curves were registered after 30 min of open circuit potential (OCP) stabilization. The potential sweep ranged from −0.3 V_OCP_ to +1.0 V_OCP_ with a scan rate of 1 mV/s. For short run times (0.5 h), the cleaned, etched and coated substrates were evaluated per triplicate and the effect of the cathodic potential (−1.0 V or −1.5 V) was compared.

EIS experiments were performed to evaluate the polarization resistance of the ZrO_2_ nanolayer, after 30 min of time exposure to the electrolyte solution. The amplitude of the EIS perturbation signal was 10 mV, the frequency ranged from 10^5^ to 10^−1^ Hz taking measurements of 10 frequencies per decade. The same cell and electrodes reported for polarization tests were used here.

## 3. Results and Discussion

### 3.1. Influence of the Substrate in the Deposition of Zr-EAD Films

There are some studies that corroborate the effective role of intermetallic particles in alloyed aluminum as promoters of Zr-CCC deposition [9,25], but apparently their effect in the Zr-EAD has not been explored yet. Pioneer works of Zr-based coatings onto aluminum substrates were also limited to CCC treatments [26,27]. In all cases, the major drawback is the high porosity of the oxide layer and the limited thickness of the ZrO_2_ film (10–80 nm), obtained by CCC processes, that preclude a good coating analysis. Therefore, this section will focus in the surface characterization of ZrO_2_ with controlled deposition by EAD method.

**Figure 1:**
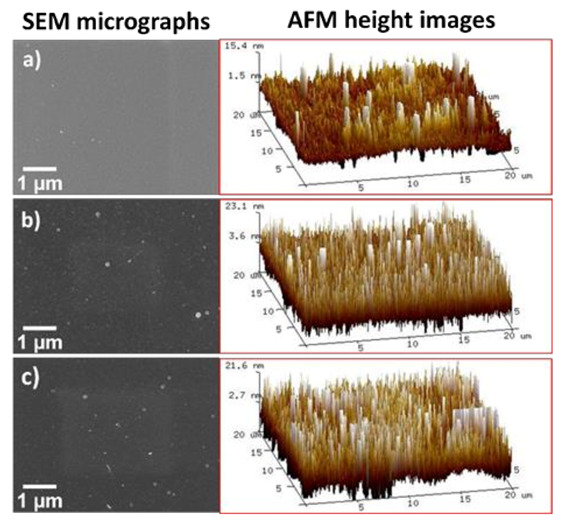
SEM (**left**) and AFM 3D height images (**right**) on ITO substrates: (**a**) after ultrasonication bath cleaning; (**b**) after Zr-EAD at −1.0 V; and (**c**) after Zr-EAD at −1.5 V.

The properties of the ZrO_2_ deposition will depend on the substrate nature and its roughness. Therefore, indium-tin oxide substrates, with very low roughness (4.3 ± 0.9 nm) were chosen to explore the deposition of ZrO_2_ nanometric layer, taking the advantage of the low effect of the substrate roughness on the nanocoating obtaining. The other advantage of ITO substrate is that it does not undergo the reactions involved in the self-deposition of the ZrO_2_ by the CCC method, i.e., the zirconium coating can only be obtained with the application of current or voltage (EAD method). As can be seen in the topographic images (Figure 1a–c), the roughness of the ZrO_2_ layer is practically the same than that of the substrate without EAD coating, independently of the potential applied. This evidenced that the formed film reproduces the underlying substrate roughness (Table S1, supporting information). The root mean square roughness (Rq) values, which were determined by AFM using a scan window size of 20 μm^2^ × 20 μm^2^, were 5.0 ± 0.4 nm, 6.6 nm ± 0.4 nm, and 6.3 nm ± 0.5 nm, respectively for ITO bare, Zr-EAD/ITO −1.0 V and Zr-EAD/ITO −1.5 V (Table S1). Increase of electrochemical reaction time (from 4 min to 60 min) led to the obtaining of thick ZrO_2_ films with elastic modulus of 14.1 ± 2.1 GPa and hardness of 1.3 ± 0.1 GPa, derived from nanoindentation measurements (Figure S1). Such values do not correspond to solid fully dense zirconia, after sintering process (>17 GPa and 250 GPa, for hardness and elastic modulus, respectively) [28], as the coating does not undergo traditional ceramic processing, indicating that the film should have more porous microstructure than that of bulk material itself [29,30,31].

**Figure 2:**
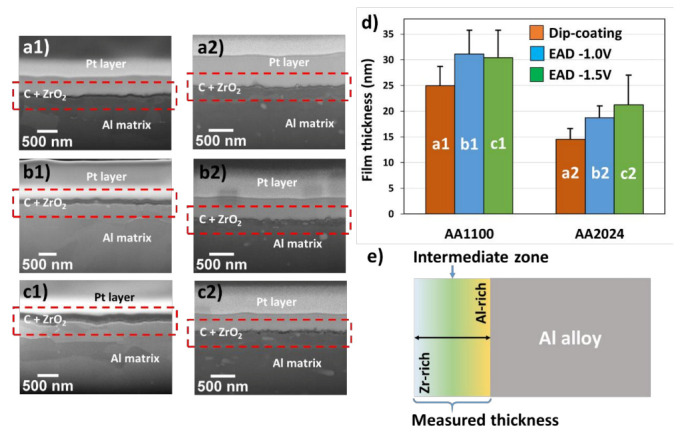
(**a**–**c**) Cross-section of FIB-SEM micrographs showing the ZrO_2_ film thickness between Pt layer and metal substrate, previously degreased with alkaline solution (Bare). (**d**) Values of thicknesses and deviations of samples AA1100 and AA2024, evaluated by HR-SEM. (**e**) Schematic representation of ZrO_2_ layer over Al alloy surfaces. Letter codification: (**a**) Dip-coating method; (**b**) Zr-EAD at −1.0 V; (**c**) Zr-EAD at −1.5 V; and number codification: (1) AA1100 and (2) AA2024 plates. SEM magnifications: (a1) 81.81 kX; (a2) 100 kX; (b1) 72.43 kX; (b2) 100 kX; (c1) 85.28 kX; and (c2) 100 kX. Red rectangles indicate the region used for the thicknesses measurements. The different layers can be easily discriminated in the Figure S2 (supporting information), and are composed by: Pt, carbon graphite and Zr-DC or Zr-EAD layers.

Regarding the deposition of ZrO_2_ in aluminum substrates, the thickness measurement by AFM is impracticable due to the nanoscale range of the films (20–30 nm) compared to the high roughness of soft metal substrate after polishing pre-treatments. Here, such measurements were carried out with FIB-SEM microscopy. The SEM images, showing a detail of the cut provoked by the electron beam, demonstrate that ZrO_2_ films are perfectly adhered to the substrate (Figure 2a–c). The substantial challenge is found when comparing the two substrates in Figure 2d. AA1100 plates have the highest film thicknesses (Table 1). The explanation is that the zirconium layer does not correspond exclusively to ZrO_2_ compounds, as the film has a gradient in composition. The closer to the substrate interface, the higher the Al concentration, which is related to a combination of Al_2_O_3_/AlOOH passivating layers; whereas the outer surface is rich on Zr atoms (detected by XPS analysis, as will be discussed in Section 3.2). This implies that there is not a defined interface between the Zr-CCC and the native oxide/hydroxide layer and both contribute to the measured thicknesses. A simplified profile in Zr and Al concentrations, as a function of depth, is shown in Figure 2e. The depth–dependent element concentration profile on zirconium-based conversion coatings has been already demonstrated by other authors using techniques such as Auger Electron Spectroscopy (AES) [17,32], Time-of-Flight Secondary Ion Mass Spectrometry (ToF-SIMS) [33] and Glow Discharge Optical Emission Spectroscopy (GDOES) [34]. The thickness of the native oxide layer was determined to be at around 30 nm.

The local deposition of Zr-oxide based coatings on AA1100 is due its high amount of passivating layers (Al_2_O_3_/AlOOH), compared to the surface with copper rich sites (AA2024). Moreover, previous work proved that the local composition of ZrO_2_ is conditioned by the presence of copper in the alloy surface (AA6014 contains Mg ≤0.8 wt.%, Fe ≤0.35 wt.%, Si ≤0.6 wt.%, Cu ≤0.2 wt.%, Mn ≤0.2 wt.%, the rest of Al) [35]. They found higher amount of Al atoms than Zr atoms when alloys without Cu were investigated (AA1050 contains Al ≥ 99.5 wt.%, Fe ≤ 0.3 wt.%); which corroborates our results. Thus, we found similar effect and the same tendency on ZrO_2_ layer deposition, by using XPS analysis (Section 3.2). The novelty in this work is that the aluminum alloy used has the highest Cu weight percentage (AA2024 contains Cu ≥ 4.5 wt.%) among Al alloys. Therefore, the effect on the nanocoating deposition is more evident with respect to samples without copper, as for example in AA1100 and AA1050 alloys; or with very low content, as is the case of AA6014 alloy.

On the other hand, the analysis of variance followed by Turkey test for means comparison (significance level of 0.05) indicates greater film thicknesses obtained by EAD, regardless of the applied potential, when compared to the DC method (Table 1) on both substrates. This probably occurs due to the improved ions migration induced by potential application.

### 3.2. Zr-EAD Nanocoating Composition

The XPS analysis (Figure S3) revealed a great contribution of some compounds present in the metal-zirconium oxide coating interface, due to the low thicknesses of such films and to the influence of metal pre-treatment. The most relevant atoms were aluminum, derived from the substrate and from oxidized Al compounds; fluorine, derived from the bath solution and further reaction with Al^3+^ (AlF_3_); and copper, derived from Cu oxidation to Cu^1+^ (Cu_2_O) and Cu^2+^ (Cu(OH)_2_). The latter was exclusively detected in the AA2024 substrates (Figure S4), which have high content of Cu as alloying element.

**Figure 3:**
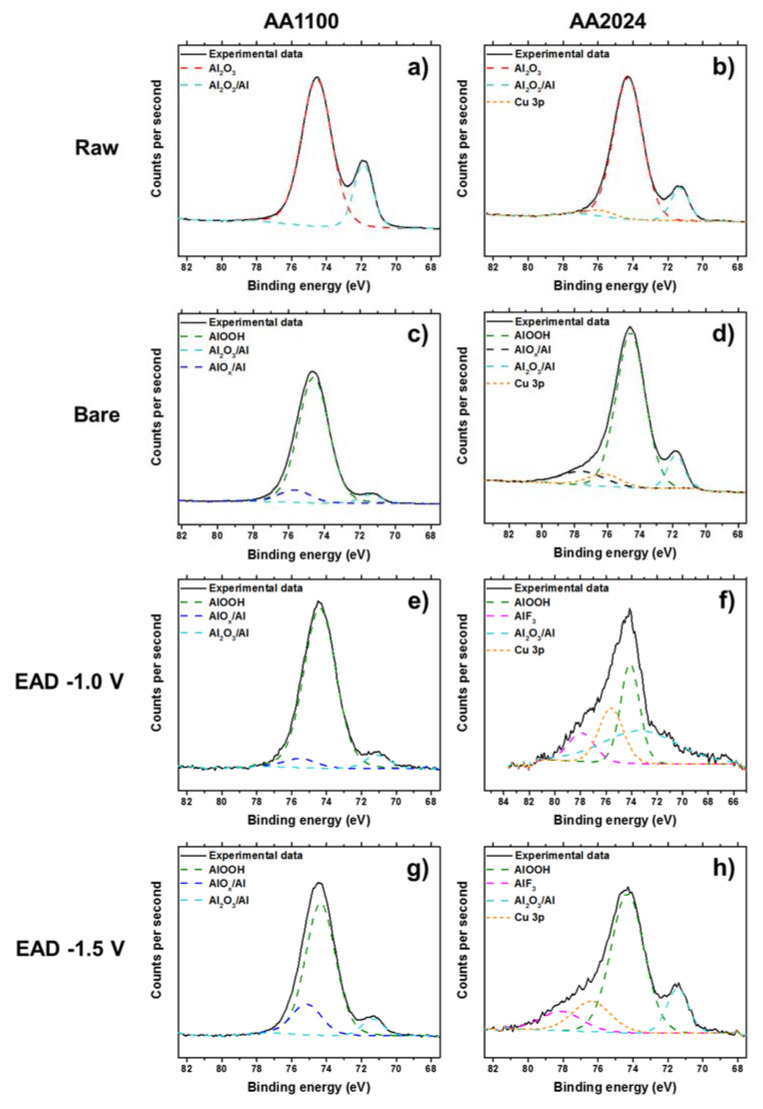
XPS high-resolution spectra of Al 2p region, before (Raw, **a**,**b**) and after alkaline etching process (Bare, **c**,**d**), and after electro-assisted deposition of ZrO_2_, generated at −1.0 V (**e**,**f**) and −1.5 V (**g**,**h**). Left: AA1100 samples and right: AA2024 samples.

**Figure 4:**
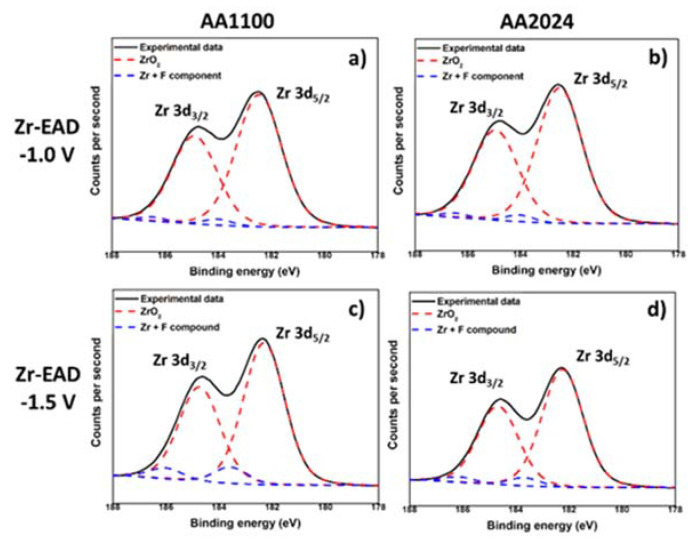
XPS high-resolution spectra of Zr 3d region, after electro-assisted deposition of ZrO_2_: (**a**) AA1100/EAD −1.0 V; (**b**) AA2024/EAD −1.0 V; (**c**) AA1100/EAD −1.5 V; and (**d**) AA2024/EAD −1.5 V.

The abovementioned compositions were observed thanks to the careful deconvolution of the XPS high resolution experimental data and contrasting with XPS database. The deconvolutions of the Al 2p, Zr 3d and Cu 2p peaks are shown in Figure 3 and Figure 4 and Figure S4. Moreover, the atomic percentage concentrations of those elements are expressed in Table S2.

It is evident that mild surface cleaning with organic solvents (Raw), for example, does not remove the native Al oxide and alloying elements from the metal surface (Figure 3a,b), whereas alkaline etching (Bare) causes a great effect. In a strongly alkaline environment, a powerful oxidizing agent is introduced, then the metal oxidation and the formation of one (or more) of the solid oxides (Al_2_O_3_/AlO_x_), and metal hydroxides (AlOOH) is expected (Figure 3c,d) [36,37]. Indeed, in the particular case of Al alloys, de-alloying of the elements close to the surface is also achieved. It was observed in the high-resolution spectra of Cu 2p, by detection of the presence of Cu_2_O and Cu(OH)_2_ molecules (Figure S4) and has been extensively reported by several authors [38,39,40,41]. Thus, the Cu atomic concentration is much higher for the Bare sample than for the other treatments in AA2024. After the zirconium deposition, the high resolution spectrum of Al 2p is almost invariable in any EAD potentials, but has much lower atomic percentage in AA2024, if compared to AA1100 (Table S2). We should emphasize that Al 2p region has also a small contribution of Cu 3p content in the same region, as seen in the Figure 3f,h, and it must be taken into account for the calculation of the Al atomic percentage in AA2024 samples.

Taking into account the deconvolution of Zr high resolution spectrum, with two peaks of Zr 3d_5/2_ and Zr 3d_3/2_ at binding energies of 182.5 eV and 185 eV (Figure 4), respectively, the inorganic coating is mainly composed by zirconium oxide, whereas some minor residual fluorine complexes are probably present [17,25,42]. Fluorine element observed in EAD samples (Figure S3) most arises from the bath, whereas that detected in Raw and Bare substrates comes from some equipment contamination. Zhang et al. [10] have reported that conversion coatings of 5052 aluminum alloy (Mg-rich alloy) obtained from fluorine salt solutions contains hydrated AlF_3_ compound trapped among the metal oxides. As the contribution of fluorine has been measured to be between 2–3%, and even less for Raw and Bare substrates, it has been considered to not interfere significantly in the interpretation of the results regarding the main components. The EAD coating composition found was consistent with that reported for DC method [25,35,42]. In AA1100 samples, the Al-rich and Zr-rich zones pointed out in Figure 2e, are not differentiated. The apparent atomic ratio of Al/Zr is close to unit (0.91 and 1.41, for EAD −1.0 V and EAD −1.5 V, respectively); whereas in AA2024 such values have been reduced to a factor of 1/3 and 1/2 (Table S2). Based on XPS evidences, undoubtedly, the unique explanation for the high content of Zr atoms in AA2024 is the distribution of ZrO_2_ molecules over the abovementioned surface, i.e., the homogeneity of the film covering the substrate, which seems to be favored by both, the use of the EAD technique and the presence of Cu-rich sites.

In conclusion, the slightly higher thicknesses measured for AA1100 samples can be the result of a thick aluminum oxide/hydroxide layer in the boundary zone between Al metal surface and zirconia layer, as explained before. So, the Zr-rich zone is expected to be more homogenous and thinner in AA2024 than in AA1100, which agrees with the known favorable effect of the intermetallic (with cathodic nature, like copper-aluminum) for the zirconium oxide film formation, described above. Additionally, comparing the two cathodic potentials used in EAD treatments, the low voltage (−1.0 V) seems to potentiate a better surface coverage, as will be explained in the next section.

### 3.3. Zr-EAD Nanocoating Surface Topography

**Figure 5:**
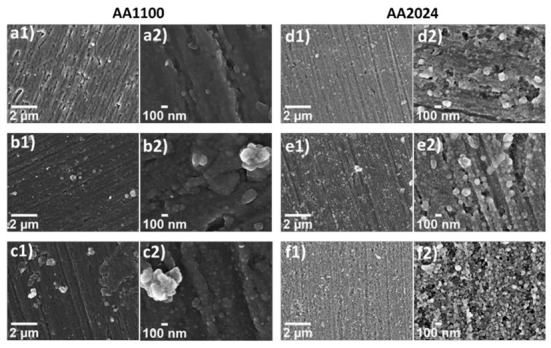
SEM micrographs at low (1, 30 kX) and high magnifications (2, 150 kX), evidencing the morphology of zirconium oxide in the following samples: (**a**) Zr-DC/AA1100; (**b**) Zr-EAD−1.0V/AA1100; (**c**) Zr-EAD −1.5 V/AA1100; (**d**) Zr-DC/AA2024; (**e**) Zr-EAD −1.0 V/AA2024; and (**f**) Zr-EAD −1.5 V/AA2024.

The morphology of the nanometric films was evaluated by SEM. In Figure 5, a clear cauliflower structures was evidenced after the ZrO_2_ growth on top of aluminium surfaces, which is characteristic of porous materials [43,44,45]. The globular agglomerates observed in samples AA1100 (Figure 5a–c) are bigger than that obtained in AA2024 (Figure 5d–f) and it affects the heterogeneity of Zr-based coatings. Long reaction time (60 min) leads to an increase on cauliflower globular structures, agglomerated and, therefore, to highly heterogeneous films with poor mechanical properties, showing thicker coating and cracks (Figure S5), not desirable for industrial application aims.

AFM was performed in order to get a deeper understanding on the surface modification by application of EAD method. The surface roughness decreased from 57.2 ± 28.4 nm in AA1100 Bare surfaces to 29.8 ± 7.4 nm and to 41.4 ± 3.8 nm, after Zr-EAD at −1.0 V and Zr-EAD at −1.5 V, respectively (Table S1, Figure S6). Similar result was obtained for AA2024 where the roughness decreased from 35.6 ± 6.6 nm to 24.9 ± 2.2 nm after Zr-EAD at −1.0 V, whereas with Zr-EAD at −1.5 V the value increased (49.4 ± 9.3 nm) respect to Bare sample (35.6 ± 6.6 nm). By contrary, in ITO substrates, the roughness differences are negligible, as expected, thanks to the high nanometric smoothness of such surfaces.

**Figure 6:**
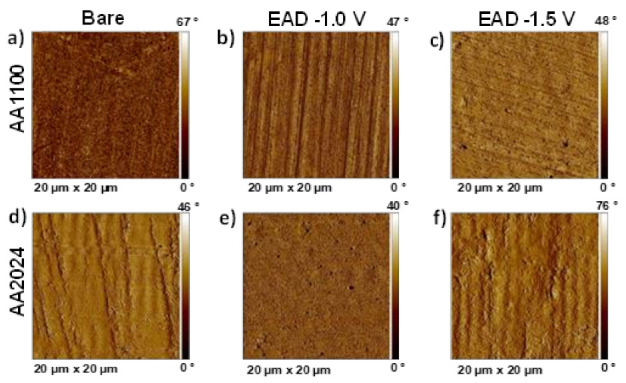
AFM 2D phase images of: (**a**) AA1100 Bare sample, after raw material degreasing; (**b**) Zr-EAD at −1.0 V/AA1100; (**c**) Zr-EAD at −1.5 V/AA1100; (**d**) AA2024 bare sample, after raw material degreasing; (**e**) Zr-EAD at −1.0 V/AA2024; (**f**) Zr-EAD at −1.5 V/AA2024.

Particularly, detail observation of AFM 2D phase images reveals important differences among pure Al and Cu-rich Al surfaces (Figure 6). ZrO_2_ successfully filled the valleys generated by the mechanical polishing marks, offering a more homogenous and smooth surface in AA2024 than in AA1100 samples (Figure 6e,f). Moreover, AFM analysis corroborated the morphology aspects observed by SEM, a high voltage (−1.5 V) implies more roughness due to the higher density of ZrO_2_ agglomerates generated. In all cases, AFM phase images presented similar and small changes in phase angle, corresponding to a homogenous coating where no more than one material is exposed on the metallic surface.

### 3.4. Wettability

**Figure 7:**
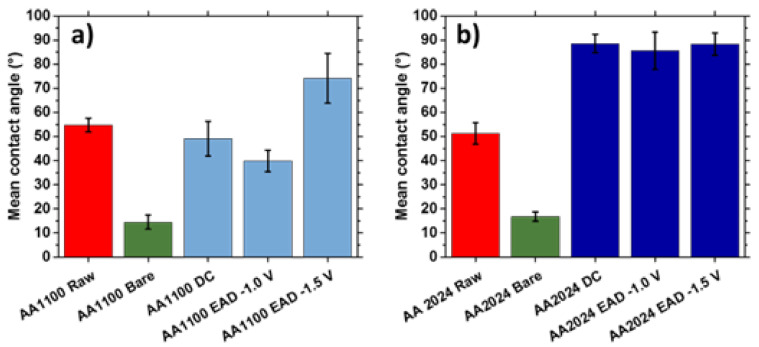
Wettability of (**a**) AA1100 and (**b**) AA2024 substrates before and after the application of the following treatments: Raw (metal plates cleaned with acetone); Bare (metal plates degreased with Saloclean 667N and grinded); DC (dip-coating method); and EAD (electro-assisted deposition at two different potentials).

The density of oxide clusters observed in Zr-EAD specimens (Figure 5) influenced both the homogeneity and the wettability of the films. The Zr-oxide films assessed in this study were hydrophilic in nature, with WCAs below 90°, as can be seen in Figure 7. This can be easily explained by comparing the different pre-treatments and the chemical nature changes provoked by each one. Raw samples have similar WCA in both AA1100 and AA2024 surfaces because the metal-air interface was not altered from etching treatments and the influence of the alloying elements is insignificant. By contrary, superhydrophilic surface was observed when the aluminum was exposed to alkaline etching (Bare samples), with WCA of 15–18° [46]. In this case, the presence of Al_2_O_3_/AlOOH passivating layers, observed either in AA1100 or in AA2024 samples (Figure 3), are the responsible for the great affinity of water molecules to this surface. Additionally, the etching of the surface with alkaline solution and the formation of oxide-hydroxide layers enhanced the roughness (Table S1) and it is another reason for their high wettability [47,48].

Moving to the zirconium oxide layers generated after conventional Zr-CCC (DC method) and comparing with Zr-EAD method, the most important changes were again observed in AA2024 samples (Figure 7b). The water affinity decreased, demonstrating that the chemical nature of this substrate plays an important role.

### 3.5. Mechanism of ZrO_2_ Deposition in AA1100 and AA2024 Surfaces

In conventional zinc phosphating pre-treatment, the initial step is a pickling attack of the metal surface by free phosphoric acid and evolution of hydrogen gas in aqueous solution. The following steps are the coating deposition (usually 2–4 µm in thickness) and the generation of high amount of sludge. Thus, replacement of zinc phosphate pre-treatments by zirconium oxide coatings is estimated to corroborate on both, the reduction of industry waste and reduction of piece weight by the deposition of an ultrathin metal oxide film (15–30 nm) [49].

**Scheme 1:**
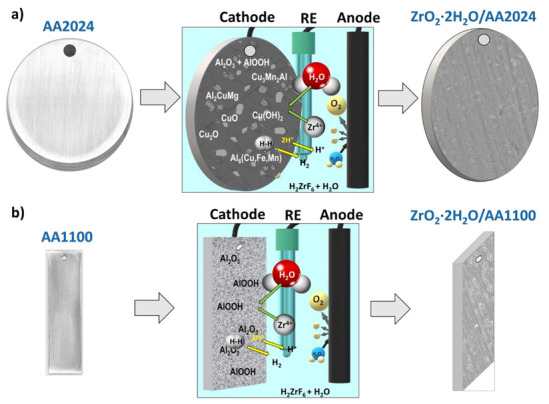
Illustration of the best conditions for Zr-EAD deposition on: (**a**) AA2024 disks and (**b**) AA1100 aluminum plates. From left to right: Bare substrates; application of −1.0 V of cathodic potential and 4 min of electrochemical reaction; Zr-EAD coated specimen. Arbitrary scale has been used for the visual representation of the reactions.

The chemical conversion coating of Zr oxide is strongly dependent on the consumption of protons [49] and on the presence of intermetallic compounds [50] on the metal surface. It is generally accepted that the first process occurring during the conversion coating formation is the thinning and partial removal of the native aluminum oxide layer by the free fluorides in the conversion bath, generating aluminum fluorides (Equation (1)), as represented in the upper part of both substrates at Scheme 1. Then, the metal surface faces a localized shift on the pH towards alkaline values due to the water electrolysis, through the generation of oxygen and consuming of protons (Equation (2)). Afterwards, the precipitation of hydrated zirconium oxides takes place, according to the reaction showed in Equation (3).

Alumina pickling:

Al^3+^_(aq)_ + ZrF_6_^2−^_(aq)_ → AlF_6_^3−^_(aq)_ + Zr^4+^_(aq)_ (1)

Water electrolysis:

2 H^+^_(aq)_ + 2e^−^ → H_2 (g)_ (2a)

H_2_O _(l)_ → ½ O_2 (g)_ + 2 H^+^_(aq)_ + 2e^−^ (2b)

Coating deposition:

Zr^4+^_(aq)_ + 4 H_2_O _(l)_ → ZrO_2_·2 H_2_O _(s)_ + 4 H^+^_(aq)_ (3)

Hydrogen evolution consumes one of the products of the fourth reaction (H^+^), that occurs in the cathode, therefore, this unbalances the reaction in the direction of products formation, enabling the production of more electro-assisted deposited coating (autocatalytic effect). Control over the cathodic potential applied (−1.0 V instead of −1.5 V) regulates the hydrogen gas generation, and thus, it can be reduced to avoid the obtaining of high porous films; which in turn is not possible with DC method.

Those reactions are particularly favored in aluminum alloys where the intermetallic particles are cathodic relative to the aluminum matrix, as other authors have already observed for the application of tri-cationic phosphating pre-treatments on AA2024 alloy [51,52]. The abundance of such cathodic sites and the preferential deposition on their surroundings is depicted in Scheme 1a. We should mention that the representation shown in Scheme 1 is a simplification of the whole process and dissolution reactions of the native oxides and hydroxides were not shown. The key aspect regarding the role of the substrate is the presence of intermetallic particles on AA2024 surfaces (Scheme 1a), which are absent in AA1100 (Scheme 1b). The presence of intermetallics and other Cu-rich particles offers a faster deposition of ZrO_2_ molecules in AA2024 substrates; and it would be one of the reasons for obtaining thicker films in such samples, as explained above. In both substrates, the porosity of the native oxide/hydroxide Al molecules also helps Zr^4+^ migration to the inner interface layer. This effect is raised by the potential application, which did not occur with conversion chemical coating deposition.

In summary, both EAD and DC techniques enable an uniform film deposition [19,53]. It has been observed that, despite the ZrO_2_ coating formation is supposedly favored by the application of low cathodic potential in electro-assisted method, the correct mechanism is probably a combination of both a chemisorption process and the electro-assisted one happening simultaneously.

### 3.6. ZrO_2_ Nanocoating Stability in Sodium Chloride Solution

**Figure 8:**
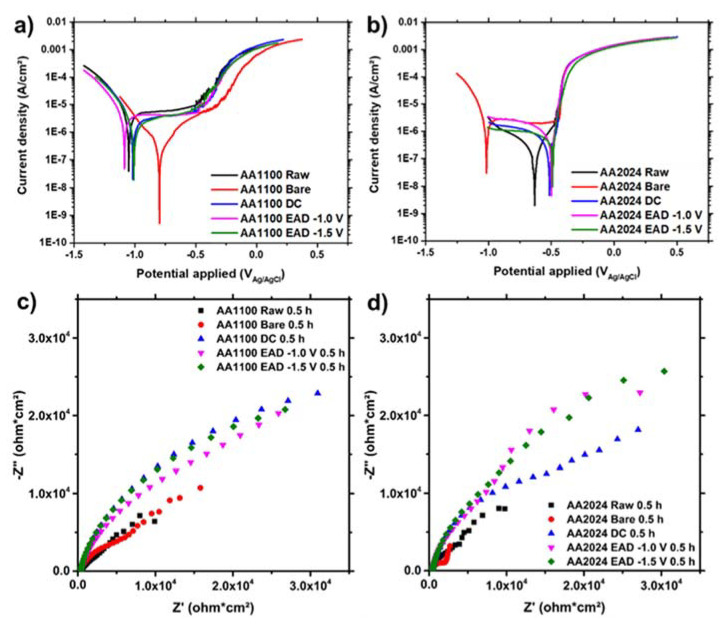
(**a**,**b**) Polarization curves and (**c**,**d**) Nyquist plots of AA1100 (**a**,**c**) and AA2024 (**b**,**d**) specimens after 30 min of immersion in aqueous NaCl 0.05 M electrolyte.

Finally, the ZrO_2_ nanocoatings, generated by DC and EAD methods, were evaluated by potentiodynamic polarization curves and EIS, after 30 min of immersion in NaCl 0.05 M for E_ocp_ stabilization (Figure 8). As can be seen, AA1100 exhibits a passivation behaviour corresponding to a contribution of a pseudo-stable interface layer in all substrates, with the exception of surfaces degreased with alkaline solution. This bare sample results in more active cathodic and anodic regions due to the growth of highly higroscopic and porous compounds, like AlOOH or Al(OH)_3_ hydroxide compositions (Figure 8a), as evidenced by XPS (Section 3.2). The measured corrosion current densities (j_corr_), corrosion potentials (E_corr_) and breakdown potentials (E_b_) for the AA1100 curves are shown in Table S3. All samples, with the exception of the bare one, display more positive breakdown potentials (E_b_) respect to E_CORR_. Conversely, AA2024 surfaces show the behavior of active corrosion, even though both the cathodic and the anodic polarization current densities decrease with respect to pure Al surfaces (Figure 8b). The less negative E_corr_ in open circuit potential, which is observed for AA2024 samples with ZrO_2_ nanocoating, and the less oxigen reduction (cathodic curves) are benefitial for this substrate compared to AA1100/ZrO_2_. Altogether allow us to conclude that ZrO_2_ layer is more compact on AA2024 than on AA1100, which is in accordance to the SEM and AFM results, as noted earlier.

Moreover, the AA2024 surface in bare samples (Figure 8b) probably has a positive barrier contribution of CuO, Cu_2_O and Cu(OH)_2_ species, which are much more stable than Al hydroxides in similar potential and pH ranges (Pourbaix diagrams). However, the E_corr_ is too low (−0.82 V) and close to the standard reduction potential of pure Al (−0.76 V). After DC and EAD ZrO_2_ depositions, the E_corr_ moves to positive values and j_corr_ values are the lowest ones (10^−7^ A/cm^2^) (Table S3).

Analyzing the DC and EAD methods for the ZrO_2_ conversion nanocoating deposition on AA1100 and AA2024 substrates, the E_corr_ and the j_corr_ are very similar for short exposition time. A detailed inspection of the effect of the the lowest cathodic potential (−1.5 V), in the coating generation compared to the highest potential (−1.0 V), proves that they also have similar electrochemical behaviour. Therefore, we conclude that the differences on the potential applied do not affect the barrier protection of the film, whereas it is mostly influenced by the substrate nature and the pre-treatments applied.

The presence of low content of copper is known to be beneficial for the formation of Zr-based conversion coatings on aluminium alloys due to the difference of potential observed between the aluminium matrix and the Cu-rich intermetallic boundaries [25,34,54,55,56]. Polarization results after DC and EAD ZrO_2_ conversion pre-treatments in pure copper plates are discussed in the supporting information (Figure S7).

The corrosion resistance is better evaluated by measurements of polarization resistances (R_p_) by EIS surface analysis. The higher the polarization resistance (i.e., the larger the Nyquist plot diameter, Figure 8c,d), the greater the barrier effect of the coating. The highest R_p_ values were found for substrates covered with the ZrO_2_ nanocoating. Table S4 indicates that the data for such samples were one order of magnitud higher than for raw and bare samples, for both AA1100 and AA2024 aluminium alloys. The resistance behaviour of ZrO_2_ generated by DC and EAD methods was similar on AA1100 plates (Figure 8c), whereas on AA2024 the first denoted the beginning of a second time constant, probably associated to a electrolyte diffusion at low frequencies (Figure 8d).

The capacitance of the double layer, measured as a constant phase element (CPE), was invariable. This is generally attributed to the surface reactivity, surface heterogeneity, and surface roughness regarding to current and potential distribution. Therefore, the Al-rich interface established in Figure 2e proves to be thin and homogenous in all samples.

These observations reinforce the understanding that the utilization of DC and EAD techniques in H_2_ZrF_6_ aqueous solution enables the deposition of the zirconium conversion coating in specific substrates, like those showed in this study for AA1100, AA2024 and ITO, whereas are prevented in others, like pure copper. Moreover, the electrochemical results evidence that such nanometric coatings do not represent a real protection barrier but rather a intermediate layer for further multilayer coatings depositions [27].

## 4. Conclusions

In this work, the control over the zirconium conversion nanocoating deposition on aluminum substrates was successfully achieved by the application of chemisorption (Zr-CCC) and electro-assisted deposition (EAD technique). The novelty of the research relies on the absence of any co-additive in the hexafluorozirconic acid aqueous solution, which represents a great advantage with respect to other bath solutions previously investigated.

The ZrO_2_ nanocoating generated by EAD or DC methods completely covers both the aluminum matrix and the intermetallic particles. One of the advantages of the electro-assisted method is the adequate control over the deposition parameters. The presence of copper alloying elements on AA2024 surface has also been proved to play an important role, favoring the ZrO_2_ deposition in this aluminum alloy and showing that the EAD method occurs simultaneously with the conventional conversion reactions.

Furthermore, the deposition of the Zr conversion coating on conductive ITO substrates can be obtained exclusively by EAD, proving that it has potential application to be explored in other conductive substrates for which Zr-CCC is prevented.

Therefore, the results obtained in this study drive forces towards the use of ZrO_2_ nanocoating as an electrochemically stable layer for future primer deposition in similar multicoated systems, like that employed with phosphating processes at industry.

## Figures and Tables

**Table 1:** Thicknesses of ZrO_2_ nanocoatings, obtained by cross-section analysis of FIB-SEM micrographs, for samples generated with two different methods. (DC and EAD), and with two different deposition potentials (−1.0 V and −1.5 V).

Substrate	ZrO_2_ Deposition Method	Thickness and SD (nm)
AA1100	DC	24.9 ± 6.7
	EAD at −1.0 V	31.1 ± 8.2
	EAD at −1.5 V	30.4 ± 9.8
AA2024	DC	14.5 ± 4.1
	EAD at −1.0 V	18.7 ± 4.0
	EAD at −1.5 V	21.2 ± 10.1

## Data Availability

The data presented in this study are available in the main text and supplementary file or upon request to the corresponding author.

## Supplementary Materials

The following are available online at https://www.mdpi.com/1996-1944/14/4/1043/s1, Figure S1: Elastic modulus and hardness as a function of nanoindenter penetration into sample surface: (a) Zr-EAD −1.0 V in ITO substrate; (b) Zr-EAD −1.0 V in AA1100 plates, (c) Zr-EAD −1.0 V in AA2024. All coatings generated at 1 h by EAD method. Hardness is given in black filled dots and elastic modulus in empty dots; Figure S2: Cross-section high-resolution SEM images of samples after in-situ Pt deposition: (a) Zr-EAD at −1.0 V/AA1100, and (b) Zr-EAD at −1.0 V/AA2024. SEM magnifications: (a) 89.51 kX; (b) 86.39 kX; Figure S3: XPS survey spectra showing the main elements found in the surfaces of Raw, Bare and Zr-EAD samples, obtained with −1.0 V and −1.5 V in: a) AA1100 plates and b) AA2024 disks; Figure S4: Cu 2p_1/2_ (956 eV) and Cu2p_3/2_ (933 ev) XPS high resolution spectra of: a) Raw, b) Bare, (c) Zr-EAD −1.0 V, and (d) Zr-EAD −1.5 V, detected in AA2024 disks; Figure S5: SEM micrographs evidencing the high density of ZrO_2_ agglomerates in AA2024 coated samples (EAD generated for 60 min at potentiostatic condition). The magnifications are: (a) 16kX; (b) 38kX; and (c) detail of the ZrO_2_ thick layer and the substrate at 26kX; Figure S6: AFM 3D height images of: (a) AA1100 bare sample, after raw material degreasing; (b) Zr-EAD at −1.0 V/AA1100; (c) Zr-EAD at −1.5 V/AA1100; (d) AA2024 bare sample, after raw material degreasing; (e) Zr-EAD at −1.0 V/AA2024; (f) Zr-EAD at −1.5 V/AA2024; Figure S7: Electrochemical and visual inspection of the tested pure copper plates in presence of hexafluorozirconic acid solution: (a) photographs of the analyzed areas after polarization assays, and b) potentiodynamic polarization curves in NaCl 0.05 M electrolyte (0.5 h); Table S1: Roughness of aluminum surfaces treated with degreasing agent (Bare) and electro-assisted deposition of zirconium conversion coating (EAD). The values were measured using AFM 3D topographic analyses (Figure S6) and were compared to ITO substrates; Table S2: Atomic concentration of Al 2p, Zr 3d, Cu 2p and O 1s, obtained from the XPS high resolution spectrum of each orbitals, and comparative apparent atomic ratios of main elements; Table S3: Corrosion potentials (E_corr_) and corrosion current densities (j_corr_) measured by Tafel fitting of the experimental curves obtained after samples immersion in NaCl 0.05 M for 30 min; Table S4: Electrochemical impedance parameters for all samples, after immersion in NaCl 0.5 M for 30 min.
