# Supplementary material for: Green Nanocoatings Based on the Deposition of Zirconium Oxide: The Role of the Substrate
Source: Materials (Basel). 2021 Feb 23;14(4):1043. doi: 10.3390/ma14041043 (PMC7926333; doi:10.3390/ma14041043)
Supplement: Supplementary file 1 [file materials-14-01043-s001.pdf]

materials MDPI

*Supplementary Information*

# Green Nanocoatings Based on the Deposition of Zirconium Oxide: The Role of the Substrate

**Vitor Bonamigo Moreira [1,2], Anna Puiggalí-Jou [1,3], Emilio Jiménez-Piqué [3,4], Carlos Alemán [1,3,5], Alvaro Meneguzzi [2] and Elaine Armelin [1,3,*]**

[1] Department of Chemical Engineering, Universitat Politècnica de Catalunya, Campus Diagonal Besòs (EEBE), C/Eduard Maristany, 10-14, Building I, 2nd floor, 08019 Barcelona, Spain; vitor.moreira@upc.edu (V.B.M.); anna.puiggali@upc.edu (A.P.-J.); carlos.aleman@upc.edu (C.A.); elaine.armelin@upc.edu (E.A.)
[2] Post-Graduation Program in Mining, Metallurgical and Materials Engineering (PPGE3M), Universidade Federal do Rio Grande do Sul (UFRGS), Av. Bento Gonçalves, 9500–91501-970 Porto Alegre, RS, Brazil; meneguzzi@ufrgs.br
[3] Barcelona Research Center for Multiscale Science and Engineering, Universitat Politècnica de Catalunya, Campus Diagonal Besòs (EEBE), C/Eduard Maristany, 10-14, Building I, basement floor, 08019 Barcelona, Spain
[4] Department of Materials Science and Engineering, Universitat Politècnica de Catalunya, Campus Diagonal Besòs-EEBE, C/Eduard Maristany, 10-14, Building I, 1st floor, 08019 Barcelona, Spain. emilio.jimenez@upc.edu (E.J.-P.)
[5] Institute for Bioengineering of Catalonia (IBEC), The Barcelona Institute of Science and Technology, Baldiri Reixac, 10-12, 08028 Barcelona, Spain
* Correspondence: elaine.armelin@upc.edu; Tel.: +34-934054447

## 3. Results and Discussion

### *3.1. Influence of the Substrate in the Deposition of Zr-EAD Films*

**Table S1.** Roughness of aluminium surfaces treated with degreasing agent (Bare) and electro-assisted deposition of zirconium conversion coating (EAD). The values were measured using AFM 3D topographic analyses (Figure S6) and were compared to ITO substrates.

| Substrate | $ZrO_2$ Deposition Method | Roughness (nm ± SD) |
|---|---|---|
| **AA1100** | Bare | 57.2 ± 28.4 |
| | EAD at -1.0V | 29.8 ± 7.4 |
| | EAD at -1.5V | 41.4 ± 3.8 |
| **AA2024** | Bare | 35.6 ± 6.6 |
| | EAD at -1.0V | 24.9 ± 2.2 |
| | EAD at -1.5V | 49.4 ± 9.3 |
| **ITO** | Bare | 5.0 ± 0.4 |
| | EAD at -1.0V | 6.6 ± 0.4 |
| | EAD at -1.5V | 6.3 ± 0.5 |

Figure S1a shows that a nearly steady plateau was reached for both hardness and elastic modulus after the nanoindenter reaches the end of the oxide layer in ITO substrates. Those values correspond to the properties of the substrate. By contrary, the nanoindentation curves obtained for AA1100 plates and AA2024 disks coated with $ZrO_2$ films show a high standard deviation (Figures S1b and S1c, respectively). This behaviour is a consequence of a relatively high surface roughness compared to the nanoscale dimensions of $ZrO_2$ coatings. As can be seen, in the Figures S1b-c, the nanoindenter experiments over aluminum plates/ $ZrO_2$ cannot be taken into consideration for the evaluation of its mechanical properties due to the very high deviations of results, which prevent any trustful conclusions in such metallic substrates.

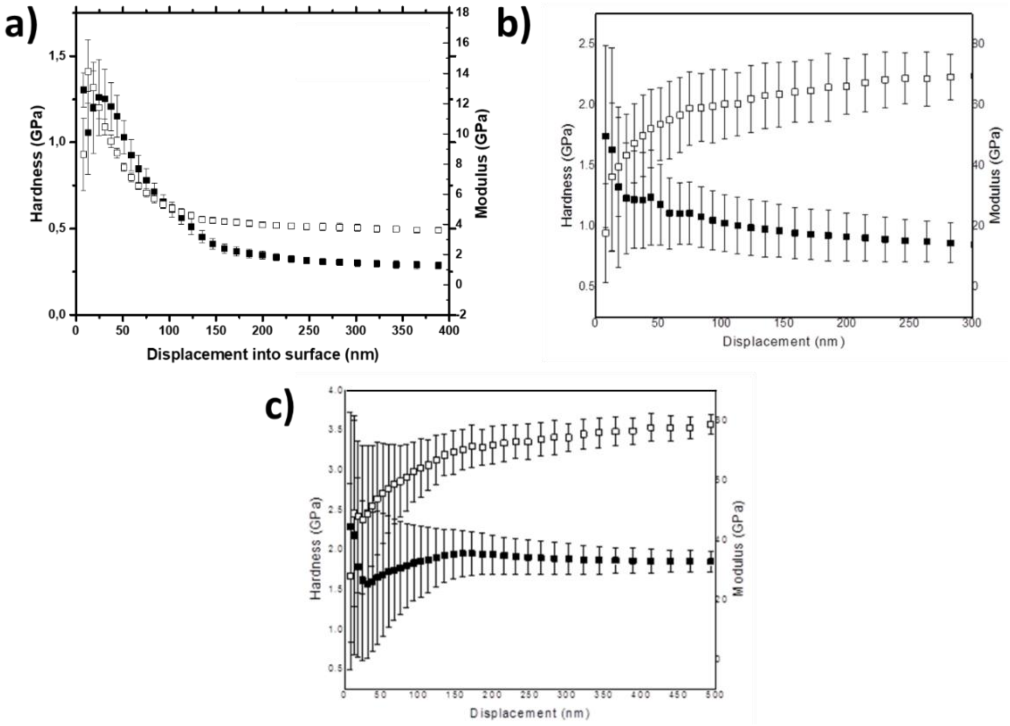


**Figure S1.** Elastic modulus and hardness as a function of nanoindenter penetration into sample surface: (a) Zr-EAD -1.0 V in ITO substrate; (b) Zr-EAD -1.0V in AA1100 plates, (c) Zr-EAD -1.0V in AA2024. All coatings generated at 1h by EAD method. Hardness is given in black filled dots and elastic modulus in empty dots.

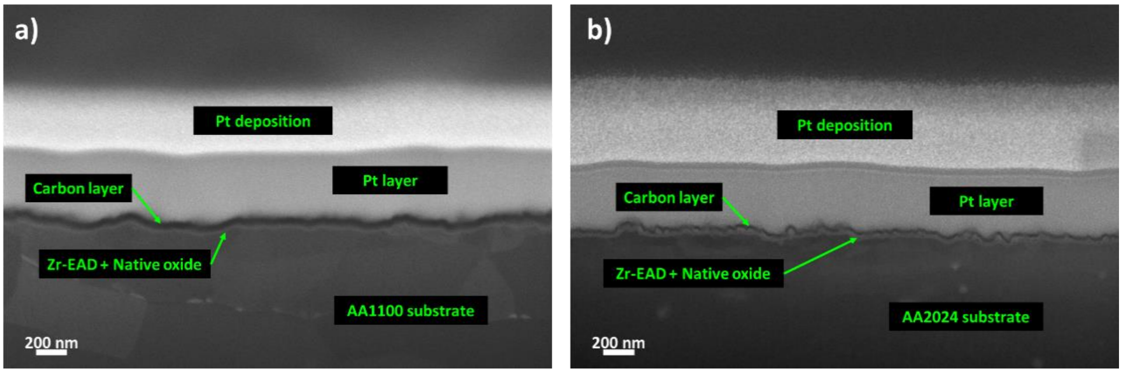


**Figure S2.** Cross-section high-resolution SEM images of samples after in-situ Pt deposition: a) Zr-EAD at -1.0 V/AA1100, and b) Zr-EAD at -1.0 V/AA2024. SEM magnifications: a) 89.51 kX; b) 86.39 kX. The $ZrO_2$ nanocoatings were covered with carbon graphite to avoid electron discharge interferences.

## *3.2. Zr-EAD Nanocoating Composition*

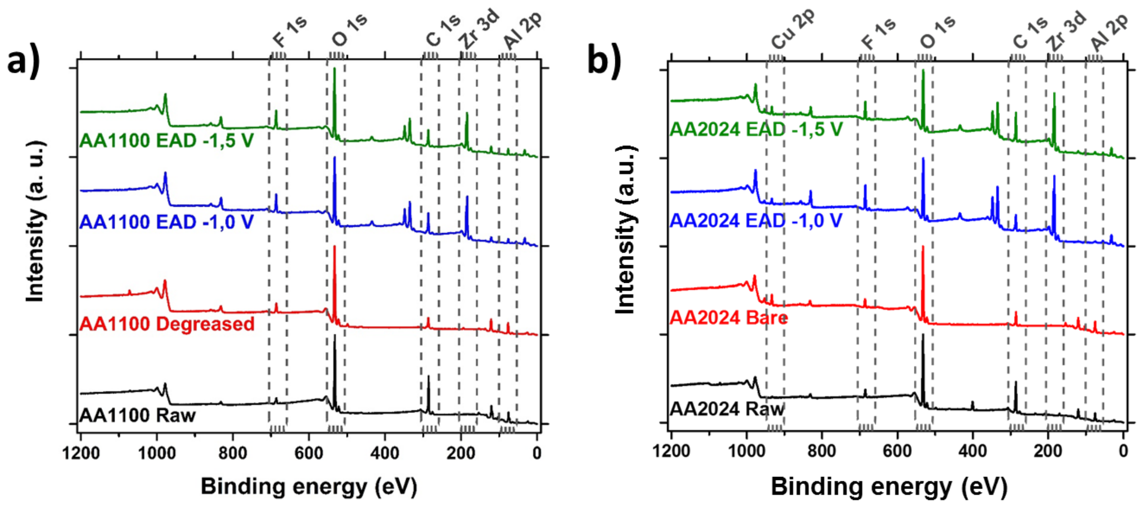


**Figure S3.** XPS survey spectra showing the main elements found in the surfaces of Raw, Bare and Zr-EAD samples, obtained with −1.0 V and −1.5 V in: a) AA1100 plates and b) AA2024 disks.

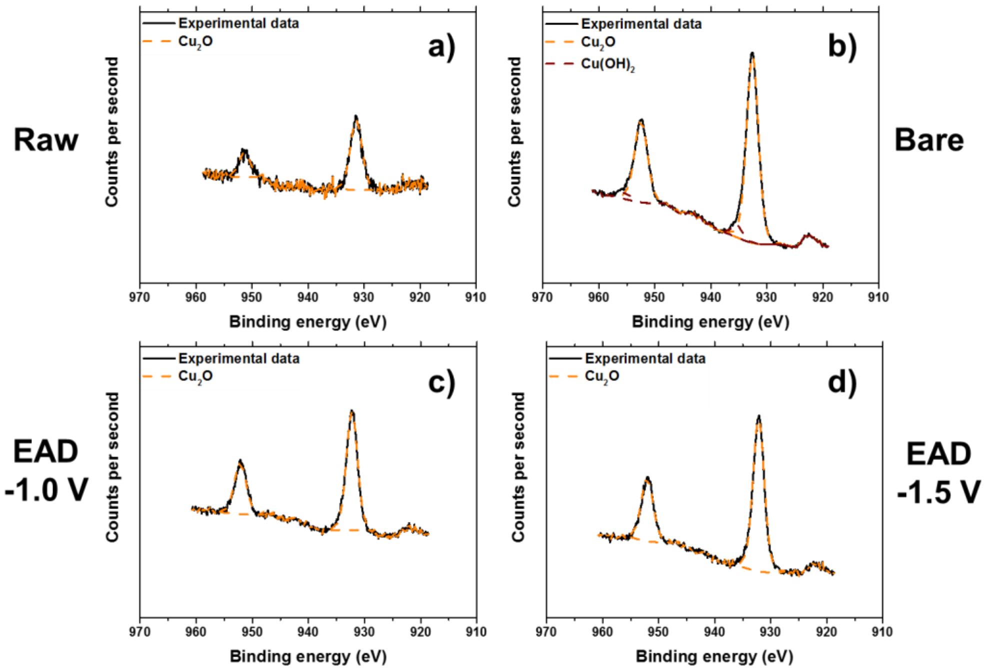


**Figure S4.** Cu 2p1/2 (956 eV) and Cu2p3/2 (933 ev) XPS high resolution spectra of: a) Raw, b) Bare, c) Zr-EAD −1.0V, and d) Zr-EAD −1.5V, detected in AA2024 disks.

**Table S2.** Atomic concentration of Al 2p, Zr 3d, Cu 2p and O 1s, obtained from the XPS high resolution spectrum of each orbitals, and comparative apparent atomic ratios of main elements. The values of Al 2p in AA2024 samples are the neat contribution of this orbital, having docked the contribution of Cu 3p from deconvoluted peaks of Al 2p region (see Figure 3, main text).

| Sample Code | Al 2p (%) | Zr 3d (%) | O 1s (%) | Cu 2p (%) | Al/Zr Ratio | Cu/Al ratio |
|---|---|---|---|---|---|---|
| **AA1100 Raw** | 27.23 | – | 27.47 | – | – | – |
| **AA1100 Bare** | 32.10 | – | 42.42 | – | – | – |
| **AA1100 EAD - 1.0 V** | 12.08 | 13.28 | 35.04 | – | 0.91 | – |
| **AA1100 EAD - 1.5 V** | 16.73 | 11.90 | 37.42 | – | 1.41 | – |
| **AA2024 Raw** | 21.25 | – | 29.70 | 0.03 | – | 0.001 |
| **AA2024 Bare** | 38.16 | – | 35.25 | 0.31 | – | 0.008 |
| **AA2024 EAD - 1.0 V** | 6.44 | 22.19 | 34.39 | 0.17 | 0.29 | 0.045 |
| **AA2024 EAD - 1.5 V** | 8.24 | 16.12 | 29.75 | 0.20 | 0.51 | 0.062 |

### *3.3. Zr-EAD Nanocoating Surface Topography*

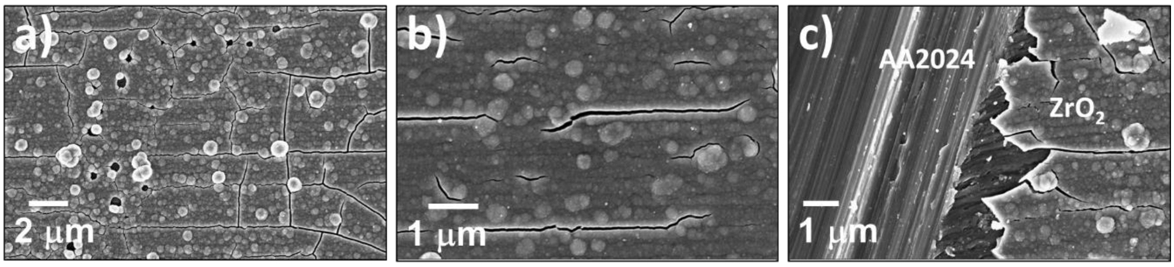


**Figure S5.** SEM micrographs evidencing the high density of $ZrO_2$ agglomerates in AA2024 coated samples (EAD generated for 60 min at potentiostatic condition). The magnifications are: (a) 16 kX; (b) 38 kX; and (c) detail of the $ZrO_2$ thick layer and the substrate at 26 kX.

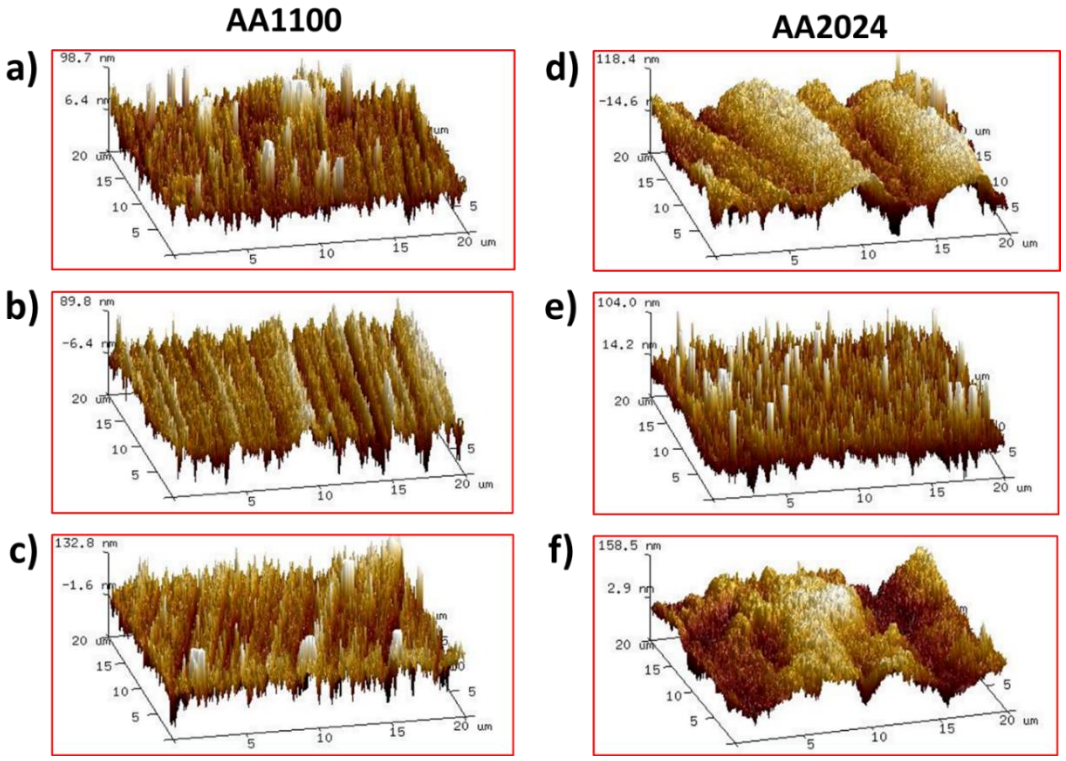


**Figure S6.** AFM 3D height images of: (a) AA1100 bare sample, after raw material degreasing; (b) Zr-EAD at −1.0 V/AA1100; (c) Zr-EAD at −1.5 V/AA1100; (d) AA2024 bare sample, after raw material degreasing; (e) Zr-EAD at −1.0 V/AA2024; (f) Zr-EAD at −1.5 V/AA2024.

### *3.6. $ZrO_2$ Nanocoating Stability in Sodium Chloride Solution*

**Table S3.** Corrosion potentials ($E_{corr}$) and corrosion current densities ($j_{corr}$) measured by Tafel fitting of the experimental curves obtained after samples immersion in NaCl 0.05M for 30 min.

| **Sample Code** | **$E_{corr}$ (V) (Value ± *SD*)** | **$J_{corr}$ (A/cm²) (Value ± *SD*)** | **$E_b$ (V) (Value ± *SD*)** |
|---|---|---|---|
| AA1100 Raw | −0.923 (*± 0.200*) | $3.35 \times 10^{-6}$ (*± $2.48 \times 10^{-6}$*) | − 0.495 (*± 0,024*) |
| AA1100 Bare | −0.851 (*± 0.055*) | $7.23 \times 10^{-7}$ (*± $3.41 \times 10^{-7}$*) | – |
| AA1100 DC | −0.997 (*± 0.012*) | $2.91 \times 10^{-6}$ (*± $4.17 \times 10^{-7}$*) | − 0.492 (*± 0.017*) |
| AA1100 EAD - 1.0 V | −1.089 (*± 0.031*) | $5.56 \times 10^{-6}$ (*± $2.11 \times 10^{-6}$*) | − 0.461 (*± 0.017*) |
| AA1100 EAD - 1.5 V | −0.995 (*± 0.009*) | $2.59 \times 10^{-6}$ (*± $2.11 \times 10^{-7}$*) | − 0.457 (*± 0.074*) |
| AA2024 Raw | −0.576 (*± 0.045*) | $4.52 \times 10^{-7}$ (*± $2.59 \times 10^{-7}$*) | – |
| AA2024 Bare | −0.802 (*± 0.268*) | $1.57 \times 10^{-6}$ (*± $9.21 \times 10^{-7}$*) | – |
| AA2024 DC | −0.517 (*± 0.033*) | $8.13 \times 10^{-6}$ (*± $2.94 \times 10^{-7}$*) | – |
| AA2024 EAD - 1.0 V | −0.575 (*± 0.145*) | $9.77 \times 10^{-6}$ (*± $7.39 \times 10^{-7}$*) | – |
| AA2024 EAD - 1.5 V | −0.518 (*± 0.042*) | $7.68 \times 10^{-7}$ (*± $4.00 \times 10^{-7}$*) | – |

The presence of low content of copper is known to be beneficial for the formation of Zr-based conversion coatings on aluminium alloys due to the difference of potential observed between the aluminium matrix and the Cu-rich intermetallic boundaries. However, only when these Cu-rich grain boundaries are exposed by adequate surface preparation the galvanic couples (grain boundary/Al matrix) may have an important effect on the surface attack by the electrolyte [6]. In the basis of such background, pure copper plates should not favour the dip-coating and EAD zirconium conversion coating deposition, like in Cu-rich Al substrates. This comparison was made using polarization curves and optical microscopy (Figure S7).

There were no evidences of $ZrO_2$ homogenous deposition (Figure S7a) and the polarization curves of the three tested Cu substrates were unaltered (Figure S7b).

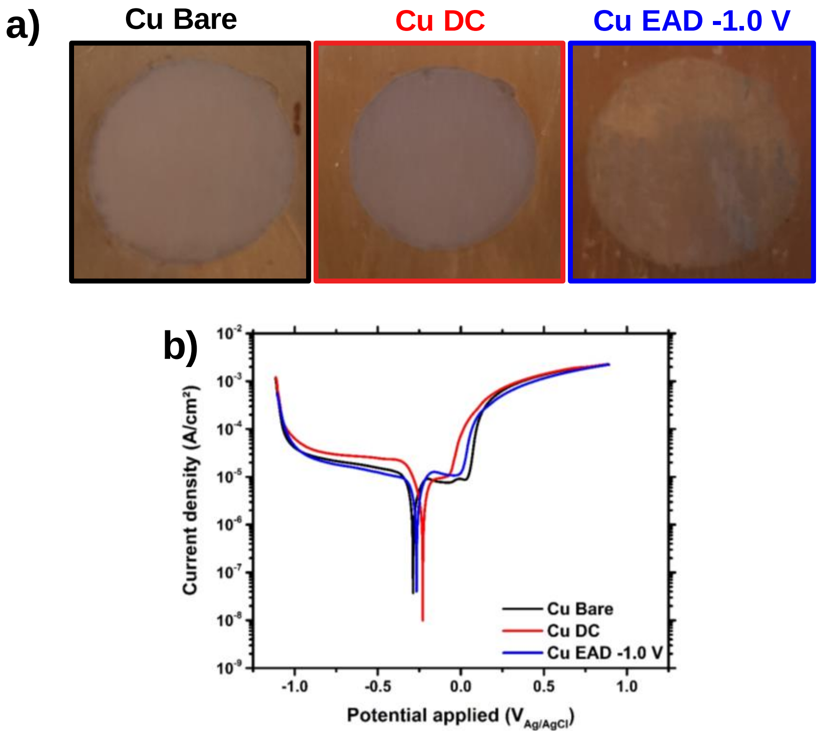


**Figure S7.** Electrochemical and visual inspection of the tested pure copper plates in presence of hexafluorozirconic acid solution: a) photographs of the analysed areas after polarization assays, and b) potentiodynamic polarization curves in NaCl 0.05 M electrolyte (0.5 h).

**Table S4.** Electrochemical impedance parameters for all samples, after immersion in NaCl 0.5M for 30 min.[a)]

| Sample Code | $R_s$ ($\Omega\cdot cm^2$) | $R_p$ ($\Omega\cdot cm^2$) | $CPE_{dl}$ ($F\cdot cm^{-2}\cdot s^{n-1}$) | $n_{CPE}$ |
|---|---|---|---|---|
| **AA1100 Raw** | 349.3 | $6.2 \times 10^3$ | $3.7 \times 10^{-5}$ | 0.695 |
| **AA1100 Bare** | 348.6 | $9.9 \times 10^3$ | $1.9 \times 10^{-5}$ | 0.798 |
| **AA1100 DC** | 220.4 | $7.0 \times 10^4$ | $2.1 \times 10^{-5}$ | 0.792 |
| **AA1100 EAD - 1.0 V** | 288.1 | $4.0 \times 10^4$ | $2.4 \times 10^{-5}$ | 0.779 |
| **AA1100 EAD - 1.5 V** | 235.3 | $5.4 \times 10^4$ | $2.9 \times 10^{-5}$ | 0.781 |
| **AA2024 Raw** | 388.8 | $5.6 \times 10^3$ | $3.9 \times 10^{-5}$ | 0.896 |
| **AA2024 Bare** | 363.0 | $2.3 \times 10^3$ | $5.7 \times 10^{-5}$ | 0.868 |
| **AA2024 DC** | 383.2 | $2.9 \times 10^4$ | $1.4 \times 10^{-5}$ | 0.855 |
| **AA2024 EAD - 1.0 V** | 394.5 | $2.7 \times 10^4$ | $2.7 \times 10^{-5}$ | 0.890 |
| **AA2024 EAD - 1.5 V** | 408.8 | $5.3 \times 10^4$ | $1.8 \times 10^{-5}$ | 0.851 |

[a]*Note*: [a)] The data were fitted using an electrical equivalent circuit (EEC) composed by one time constant $R_s(R_pCPE_{dl})$, where $R_s$ represents the ohmic resistance between the working and the reference electrodes, $R_P$ is the polarization resistance and CPE corresponds to the constant phase element of the double-layer capacitance.

## References:

1. Cerezo, J.; Taheri, P.; Vandendael, I.; Posner, R.; Lill, K.; de Wit, J.H.W.; Mol, J.M.C.; Terryn, H. Influence of surface hydroxyls on the formation of Zr-based conversion coatings on AA6014 aluminum alloy. *Surf. Coatings Technol.* **2014**, *254*, 277–283.
2. Cerezo, J.; Vandendael, I.; Posner, R.; Lill, K.; de Wit, J.H.W.; Mol, J.M.C.; Terryn, H. Initiation and growth of modified Zr-based conversion coatings on multi-metal surfaces. *Surf. Coatings Technol.* **2013**, *236*, 284–289.
3. Sarfraz, A.; Posner, R.; Lange, M.M.; Lill, K.; Erbe, A. Role of Intermetallics and Copper in the Deposition of ZrO 2 Conversion Coatings on AA6014. *J. Electrochem. Soc.* **2014**, *161*, C509–C516.

4. Šekularac, G.; Kovač, J.; Milošev, I. Prolonged protection, by zirconium conversion coatings, of AlSi7Mg0.3 aluminium alloy in chloride solution. *Corros. Sci.* **2020**, *169*, 108615.
5. Adhikari, S.; Unocic, K.A.; Zhai, Y.; Frankel, G.S.; Zimmerman, J.; Fristad, W. Hexafluorozirconic acid based surface pretreatments: Characterization and performance assessment. *Electrochim. Acta* **2011**, *56*, 1912–1924.
6. Van Ingelgem, Y.; Hubin, A.; Vereecken, J. Investigation of the first stages of the localized corrosion of pure copper combining EIS, FE-SEM and FE-AES. *Electrochim. Acta* **2007**, *52*, 7642–7650.
